# Genetically engineered transfusable platelets using mRNA lipid nanoparticles

**DOI:** 10.1126/sciadv.adi0508

**Published:** 2023-12-01

**Authors:** Jerry Leung, Colton Strong, Katherine E. Badior, Madelaine Robertson, Xiaowu Wu, Michael A. Meledeo, Emma Kang, Manoj Paul, Yusuke Sato, Hideyoshi Harashima, Andrew P. Cap, Dana V. Devine, Eric Jan, Pieter R. Cullis, Christian J. Kastrup

**Affiliations:** ^1^ Michael Smith Laboratories, University of British Columbia, Vancouver, V6T 1Z4, Canada.; ^2^ Department of Biochemistry and Molecular Biology, University of British Columbia, Vancouver, V6T 1Z3, Canada.; ^3^ Centre for Blood Research, University of British Columbia, Vancouver, V6T 1Z3, Canada.; ^4^ NanoMedicines Research Group, University of British Columbia, Vancouver, V6T 1Z3, Canada.; ^5^ Blood Research Institute, Versiti, Milwaukee,WI 53226, USA.; ^6^ Blood and Shock Resuscitation Program, United States Army Institute of Surgical Research, JBSA-FT Sam Houston, San Antonio, TX 78234, USA.; ^7^ Department of Pathology and Laboratory Medicine, University of British Columbia, Vancouver, V6T 2B5, Canada.; ^8^ Laboratory for Molecular Design of Pharmaceutics, Faculty of Pharmaceutical Sciences, Hokkaido University, Hokkaido, 060-0812, Japan.; ^9^ Centre for Innovation, Canadian Blood Services, Vancouver, V6T 1Z3, Canada.; ^10^ Departments of Surgery, Biochemistry, Biomedical Engineering, and Pharmacology and Toxicology, Medical College of Wisconsin, Milwaukee, WI 53226, USA.

## Abstract

Platelet transfusions are essential for managing bleeding and hemostatic dysfunction and could be expanded as a cell therapy due to the multifunctional role of platelets in various diseases. Creating these cell therapies will require modifying transfusable donor platelets to express therapeutic proteins. However, there are currently no appropriate methods for genetically modifying platelets collected from blood donors. Here, we describe an approach using platelet-optimized lipid nanoparticles containing mRNA (mRNA-LNP) to enable exogenous protein expression in human and rat platelets. Within the library of mRNA-LNP tested, exogenous protein expression did not require nor correlate with platelet activation. Transfected platelets retained hemostatic function and accumulated in regions of vascular damage after transfusion into rats with hemorrhagic shock. We expect this technology will expand the therapeutic potential of platelets.

## Teaser

A platelet cell therapy was created from authentic platelets collected from blood donors and then genetically modified with mRNA.

## INTRODUCTION

Platelets are an integral component of hemostasis and are routinely transfused to restore hemostatic balance in thrombocytopenic or actively bleeding patients, with approximately 2 million platelet units transfused each year in the United States (*1*). Beyond these indications, platelets could be expanded as cell therapies for other diseases because they contribute to sepsis, inflammation and arthritis (*2*, *3*), and cancer metastasis (*4*), along with targeting and retention at many diseased tissues (*5*). To create new platelet cell therapies, platelets will need to be genetically modified to express therapeutic proteins. However, a major barrier to developing new platelet cell therapies is that there are currently no methods to genetically modify donor platelets. Neither electroporation, viral vectors, lipid nanoparticles (LNP), nor commercial transfection agents have edited donor platelets to express exogenous proteins.

Indirect approaches exist that express exogenous protein in platelets or platelet-like particles by targeting platelet precursor stem cells with lentiviral vectors. Circulating platelets can be permanently modified through a bone marrow transplant of genetically modified megakaryocytes, for example, to correct hemophilia A (*6*–*8*). Platelet-like particles can be cultured from stem cells, which produce low quantities of cells that have been transfused into people (*9*, *10*). In contrast, authentic platelets collected from blood donors and intended for transfusion are available in large quantities and retain important functions of circulating platelets (*11*). Functionally modifying these authentic, donor-derived platelets would be required for most applications of platelet cell therapies.

Platelets are challenging cells to genetically modify for several reasons. They are anucleate and thus unamenable to DNA-based transfection. Platelets have the cellular machinery to synthesize proteins from mRNA and are translationally active; however, they exhibit low rates of protein synthesis compared to nucleated mammalian cells (*12*). Platelets are also easily activated in response to stress, an often nonreversible cellular state promoting blood coagulation, accompanied by an increase in cell surface expression of CD62P (*13*). An ideal transfection agent for platelets would not activate them but would preserve platelet capacity to activate when exposed to agonists.

LNP represent a potentially ideal transfection agent, due to their ability to facilitate delivery and intracellular release of genetic material. Previous attempts to transfect platelets with LNP containing mRNA (mRNA-LNP) demonstrated that mRNA delivery into platelets is possible; however, protein expression was not observed (*14*). Advancements in LNP technology have substantially improved LNP potency and efficacy, as highlighted by the first US Food and Drug Administration (FDA)–approved RNA interference therapeutic for hereditary transthyretin amyloidosis (hATTR) (*15*), as well as the mRNA-based severe acute respiratory syndrome coronavirus 2 (SARS-CoV-2) vaccines that have been received by over 2.5 billion people (*16*).

Here, we report that mRNA-LNP are capable of directly transfecting donor platelets to express exogenous proteins under specific conditions. Platelets modified with mRNA-LNP maintained their function, including accumulating locally in wounds and contributing to hemostasis after transfusion into coagulopathic rats.

## RESULTS

### LNP uniquely enable exogenous protein expression in platelets

**Fig. 1.**
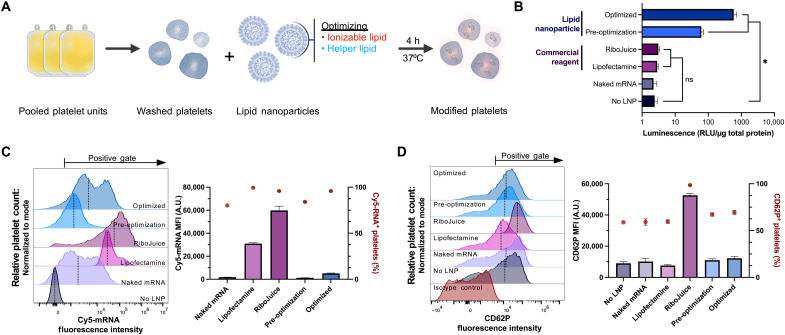
Platelets transfected with mRNA using LNP can express exogenous protein. (**A**) Schematic describing the transfection of platelets using mRNA-LNP. (**B**) NanoLuc expression, measured as the relative luminescence units (RLU) per total protein, using various transfection agents (*n* = 3). (**C** and **D**) Representative flow cytometry plots and quantification of median fluorescence intensity (MFI) (bars, left *y* axis) and percentage of platelets (red circles, right *y* axis) positive for Cy5-labeled mRNA (C) and platelet activation marker CD62P (D). The vertical dashed lines represent the MFI, and the arrows (top) represent the gate for positive events (*n* = 3). *P* values were determined by one-tailed unpaired Student’s or Welch’s *t* test. Values are mean ± SEM. ns, not significant; *P < 0.05. A.U., arbitrary units.

To identify effective transfection methods for platelets, mRNA (5′ capped and polyadenylated) encoding the enzyme NanoLuc luciferase (NanoLuc) was delivered by several transfection agents, and the expression of NanoLuc was measured (Fig. 1A). NanoLuc was not detected in platelets treated with free mRNA without a transfection agent nor by using commercial mRNA delivery reagents RiboJuice and Lipofectamine MessengerMAX, although commercial reagents enabled large amounts of mRNA to be taken up into platelets (Fig. 1, B and C). NanoLuc expression was detected using an mRNA-LNP formulation resembling the small interfering RNA–LNP that is clinically approved to treat hATTR (*17*). It consists of DLin-MC3-DMA (ionizable lipid), 1,2-distearoyl-*sn*-glycero-3-phosphocholine (DSPC; a “structural phospholipid” or “helper” lipid), cholesterol, and 1,2-dimyristoyl-rac-glycero-3-methoxypolyethylene glycol-2000 (DMG-PEG_2000_, a “PEG lipid”), hereafter referred to as MC3 DSPC. While MC3 DSPC enabled NanoLuc expression, an LNP with optimized lipid components consisting of ALC-0315 as the ionizable lipid and 1,2-dioleoly-*sn*-glycero-3-phosphocholine (DOPC) as the helper lipid (optimized) enabled a fourfold increase in uptake of mRNA-LNP and ninefold higher NanoLuc expression. The global translation inhibitor cycloheximide inhibited NanoLuc expression but not mRNA uptake, consistent with de novo protein synthesis in the mRNA-LNP–transfected platelets (fig. S1). NanoLuc expression also did not correlate with the concentration of residual white blood cells in the platelet concentrate (PC) remaining after leukoreduction, demonstrating that any contaminating leukocytes do not account for the luminescence (fig. S2). The amount of platelet activation following mRNA-LNP transfection was comparable to untreated controls that were not stimulated with agonist, measured by surface CD62P levels, whereas platelets transfected with RiboJuice had substantially increased activation (Fig. 1D). As CD62P levels can sometimes fluctuate (*18*), we also assessed the extent of activation as measured by annexin V binding to exposed phosphatidylserine. Annexin V binding was not substantially changed by transfection with mRNA-LNP (fig. S3). When additional steps were taken to minimize platelet activation during centrifugation and washing, including using platelet inhibitor prostaglandin E1 (PGE1), platelets maintained the ability to express NanoLuc. NanoLuc expression was robust while just approximately 15 and 25% of platelets were positive for CD62P with and without PGE1, respectfully (fig. S4). While the largest contribution to platelet activation was from centrifuging and washing rather than mRNA-LNP, we compared platelets transfected without PGE1 to cold-stored platelets, which are an approved product for transfusion and resuscitation in bleeding patients. Compared to cold-stored platelets, mRNA-LNP platelets displayed less CD62P and comparable CD42b (figs. S5 and S6).

### Platelet-optimized mRNA-LNP enable maximal exogenous protein expression

**Fig. 2.**
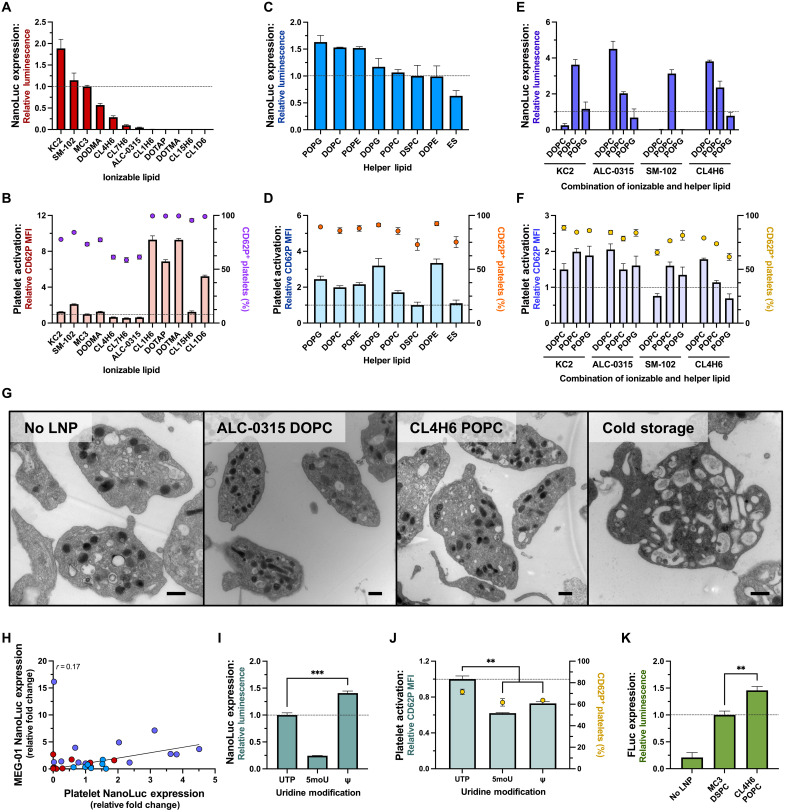
Luciferase expression and platelet activation depend on lipid composition and mRNA base modifications. (**A** to **F**) Relative NanoLuc expression and platelet activation, respectively, with (A and B) various ionizable lipids, (C and D) helper lipids, and (E and F) combinations of select ionizable and helper lipids (*n* = 3). (**G**) Transmission electron micrographs of washed platelets (WPs) without LNP (no LNP), treated with mRNA-LNP (ALC-0315 DOPC and CL4H6 POPC) and stored at 4°C (cold storage). Scale bars, 500 nm. (**H**) Correlation between the NanoLuc expression in MEG-01 cells and platelets. Colors represent screens of ionizable lipid (red), helper lipid (blue), combinations of ionizable and helper lipids (purple). (**I** and **J**) Relative NanoLuc expression (I) and platelet activation (J) with unmodified uridine base [uridine 5′-triphosphate (UTP)] or the uridine base modifications 5-methoxyuridine (5moU) or pseudouridine (Ψ) (*n* = 3). All values were normalized to MC3 DSPC (dashed line). (**K**) Relative firefly luciferase expression normalized to MC3 DSPC. *P* values were determined by one-tailed unpaired Student’s *t* test. Values are mean ± SEM. **P < 0.01; ***P < 0.001.

To identify the mRNA-LNP formulation most suitable for transfecting platelets, we optimized three major mRNA-LNP lipid components: the ionizable lipid, the helper lipid, and the PEG lipid. The ionizable lipid can markedly affect the morphology and potency of the LNP (*19*). Ten ionizable and two permanently cationic lipids were screened including clinically approved lipids ALC-0315 (*20*) and SM-102 (*21*) used in SARS-CoV-2 vaccines; previously established ionizable lipids (KC2 and DODMA) (*22*); a series of other ionizable lipids effective at transfecting many cell types (CL1D6, CL1H6, CL4H6, CL7H6, and CL15H6) (*23*); and permanently cationic lipids 1,2-di-O-octadecenyl-3-trimethylammonium propane (DOTMA) and 1,2-dioleoyl-3-trimethylammonium propane (DOTAP) (Fig. 2, A and B). Protein expression, mRNA uptake, and activation were measured and normalized to MC3 DSPC. The highest protein expression was achieved with lipids KC2, SM-102, and MC3 and intermediate expression with DODMA and CL4H6. Although lipids CL1H6, CL15H6, CL1D6, DOTMA, and DOTAP all yielded high levels of mRNA uptake (fig. S7), low protein expression and high platelet activation made these undesirable transfection reagents. ALC-0315, which is a potent ionizable LNP in most settings (*24*, *25*), gave low expression. Apart from KC2, no ionizable lipid in combination with DSPC achieved higher protein expression than MC3, and only lipids with an acid dissociation constant (pKa) between 6.5 and 6.7 enabled NanoLuc expression when transfected at a pH of 6.5 (fig. S8). Unexpectedly, lipids supporting the greatest RNA uptake did not have the highest protein expression. While the highest protein expression occurred with KC2, lipid CL4H6 induced minimal platelet activation while still supporting protein synthesis.

Helper lipids are another component which can greatly affect the structure and stability of the final mRNA-LNP (*19*). Eight different helper lipids were screened, using MC3 as the constant ionizable lipid (Fig. 2, C and D). All helper lipids tested enabled NanoLuc expression, ranging from 0.75- [egg sphingomyelin (ES)] to 1.6- [1-palmitoyl-2-oleoyl-sn-glycero-3-phospho-(1'-rac-glycerol) (POPG)] fold differences when compared to MC3 DSPC. While there were variations in mRNA uptake and activation, these differences were more subtle than those in the ionizable lipid screen. Substituting DSPC with DOPE, POPC, DOPG, or ES did not notably increase NanoLuc expression levels when formulated with MC3. POPC was identified as an optimal helper lipid, as it yielded the second lowest increase in surface CD62P median fluorescence intensity (MFI) while enabling protein expression comparable to DSPC.

To determine whether combinations of ionizable and helper lipids would have a synergistic effect in enhancing protein expression while minimizing platelet activation, we examined the two FDA-approved ionizable lipids (ALC-0315 and SM-102), as well as KC2 and CL4H6, in a combinatorial screen with the best performing helper lipids (POPG, DOPC, and POPC) (Fig. 2, E and F). mRNA-LNP formulations ALC-0315 DOPC and CL4H6 POPC yielded high protein expression, with CL4H6 POPC leading to minimal platelet activation. Expression of NanoLuc using ALC-0315 DOPC was also detected as early as 2 hours and for up to 40 hours posttransfection (fig. S9) and did not appear to substantially affect the morphological structure of the platelets compared to washed platelets (WPs) alone when assessed by transmission electron microscopy (TEM) (Fig. 2G). When tested in the commonly used immortalized platelet precursor cell line, MEG-01, these formulations yielded low levels of NanoLuc expression, and there was no correlation in relative expression between all mRNA-LNP tested in platelets and MEG-01 cells (Pearson correlation coefficient, *r* = 0.17) (Fig. 2H and fig. S10). In addition, changing the molar percentage of DMG-PEG_2000_, which is known to influence particle size and transfection potency (*19*), did not significantly influence NanoLuc expression but changed mRNA uptake and platelet activation in both ALC-0315 DOPC and CL4H6 POPC formulations (fig. S11 and table S1).

In addition to the lipid composition, mRNA elements can play a substantial role promoting efficient exogenous protein synthesis (*26*). Modified mRNA bases, such as pseudouridine and 5-methoxyuridine (5moU), are frequently used in mRNA-based therapeutics, influencing both translation and immunogenicity (*27*). We examined the effects of these modified uridine bases on platelet translation and activation. Pseudouridine-containing mRNA (Ψ) significantly increased protein expression compared to unmodified uridine, while 5moU yielded less protein expression (Fig. 2I). Both modifications significantly reduced the extent of platelet activation compared to unmodified mRNA (Fig. 2J). The cumulative effects of optimizing LNP lipids and mRNA modifications enabled the expression of firefly luciferase (Fig. 2K). Thus, LNP containing helper lipids with a phosphocholine head, along with lipids containing branched or unsaturated tail groups, were most suited for transfecting platelets and driving higher expression levels. Of the RNA modifications tested, unmodified uridine or pseudouridine was optimal for enabling higher NanoLuc expression levels.

### Exogenous protein expression in platelets is not dependent on activation

**Fig. 3.**
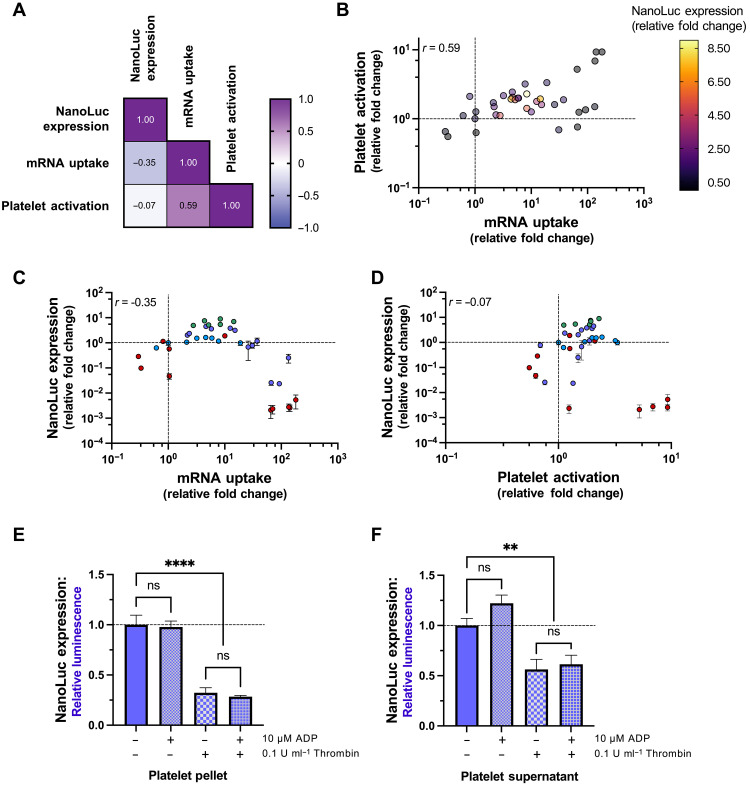
Expression of exogenous protein does not correlate with activation nor extent of mRNA uptake. (**A** and **B**) Pearson correlation matrix between relative NanoLuc expression, mRNA uptake, and platelet activation (A) with corresponding three-dimensional plot (B). (**C** and **D**) The data from (A) and (B) are replotted in two-dimensional graphs. Correlation between NanoLuc expression and mRNA uptake (C) and platelet activation (D). The dashed lines represent MC3 DSPC. Colors represent screens of ionizable lipid (red), helper lipid (blue), combinations of ionizable and helper lipids (purple), and DMG-PEG_2000_ content (green). (**E** and **F**) Relative NanoLuc expression in the platelet pellet (E) and supernatant (F) following stimulation with agonists of platelet activation. All values were normalized to CL4H6 POPC treated platelets without agonists, represented by the dashed line (*n* = 3). *P* values were determined by one-way analysis of variance (ANOVA). Values are mean ± SEM. **P < 0.01; ****P < 0.0001.

Expression of endogenous platelet proteins can occur following activation (*12*, *28*), and we initially expected that expression of exogenous proteins from transfected mRNA may also depend on activation. To determine whether the expression of NanoLuc depended on the degree of platelet activation or the amount of RNA delivered, a correlation matrix analysis on the library of mRNA-LNP tested was performed. NanoLuc expression did not correlate strongly with either surface platelet CD62P levels or mRNA uptake (Fig. 3, A and B). There was a mild positive correlation between the amount of RNA delivered and platelet activation (Pearson correlation coefficient, *r* = 0.59) (Fig. 3B), a weak negative correlation between NanoLuc expression and mRNA uptake (*r* = −0.35) (Fig. 3C), and no correlation between NanoLuc expression and surface platelet CD62P levels (*r* = −0.07) (Fig. 3D).

We next tested whether NanoLuc expression would be influenced when platelets are activated using agonists before or following mRNA-LNP transfection. Platelets stimulated with adenosine diphosphate (ADP), cross-linked collagen-related peptide (CRP-XL), or thrombin for 30 min before mRNA-LNP treatment had significantly less NanoLuc expression when assessed at 4 hours (fig. S12). Similarly, when platelets were first incubated with mRNA-LNP for 3.5 hours and then stimulated with ADP, CRP-XL, or thrombin for 30 min, platelets also did not have increased NanoLuc expression (fig. S13). Platelets undergo substantial rearrangement of their transcriptome and proteome when they are stimulated with agonists for up to 2 hours (*29*). We thus also activated platelets for 2 hours with either ADP or thrombin following 2 hours of mRNA-LNP treatment. The luminescence of both the cell pellet and supernatant fraction were measured to account for any NanoLuc potentially released during activation. There was no significant change in the expression of NanoLuc when platelets were stimulated with ADP (Fig. 3, E and F). Conversely, thrombin led to an approximately threefold decrease in NanoLuc in both fractions. Together, and unexpectedly, these results showed that translation of the exogenous mRNA did not require platelet activation, the highest expressing mRNA-LNP are those that do not induce activation, and stimulation with a potent agonist may be detrimental to exogenous protein synthesis over a period of several hours.

### Platelets treated with mRNA-LNP maintain hemostatic function in vitro

**Fig. 4.**
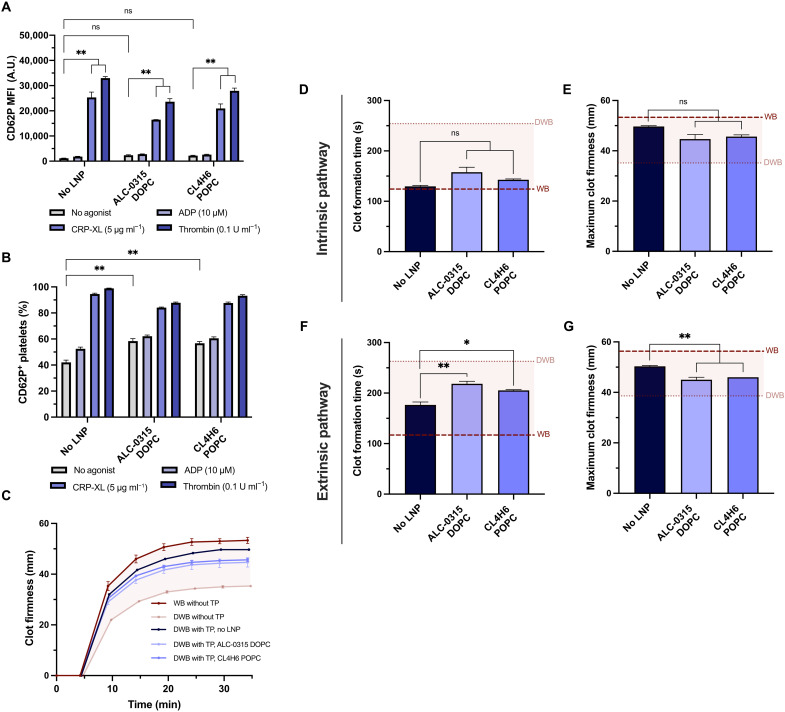
Platelets treated with LNP maintain their ability to activate and contribute to the growth and firmness of blood clots. (**A** and **B**) Platelet activation without agonists or stimulation by ADP, CRP-XL, or thrombin, as measured by the MFI of surface CD62P levels (A) and percentage of platelets positive for surface CD62P (B). (**C**) Representative ROTEM curves, with clotting initiated by ellagic acid (*n* = 3). The red shaded region is the area between whole blood (WB) and diluted WB (DWB) without the additional transfusion package (TP) added. (**D** to **G**) Quantifying ROTEM clot formation time (s) and maximum clot firmness (mm), for clotting activated with ellagic acid via the intrinsic pathway (C and D) or thromboplastin via the extrinsic pathway (E and F). The dashed lines represent firmness of WB (dark red) and DWB (light gray). *P* values were determined by one-way ANOVA. Values are mean ± SEM. *P < 0.05; **P < 0.01.

Platelets are highly sensitive to their physical and chemical environments, which adds to the challenge of genetic modification. To determine whether platelets could still be activated following mRNA-LNP transfection, their activation state and response to physiological agonists ADP or thrombin were measured. Platelets transfected with LNP responded similarly to platelets that were not transfected (Fig. 4, A and B). Without agonists, there was no significant increase in the MFI but statistically significant increase in the percentage of platelets positive for CD62P. ADP mildly increased CD62P levels, while CRP-XL and thrombin substantially increased CD62P levels in platelets transfected with mRNA-LNP.

To test whether transfected platelets retained their ability to contribute to the firmness and rate of clot formation, we used rotational thromboelastometry (ROTEM) and an ex vivo model testing platelet activity in whole blood (WB) (Fig. 4C). Diluted WB was used to model dilutional coagulopathy, a defect in hemostasis often occurring following trauma or severe bleeding with resuscitation, which increases mortality risk (*30*). Platelets were prepared in a “transfusion package” (TP), after transfecting them with LNP (ALC-0315 DOPC or CL4H6 POPC, without mRNA) and mixing with plasma and red blood cells at a clinical ratio of 1:1:1. This TP was then added to diluted WB to model transfusion into a patient with dilutional coagulopathy, and its impact on hemostasis was compared to normal WB without dilutional coagulopathy. Platelets transfected with LNP responded similarly to untreated platelets when coagulation was initiated via the intrinsic pathway of coagulation, although there was some reduction in coagulability when activated via the extrinsic pathway (Fig. 4, D to G, and tables S2 and S3). Platelets transfected with LNP significantly improved the maximum clot firmness and clot formation time of diluted WB compared to diluted WB alone when coagulation was initiated through either the intrinsic and extrinsic pathways, highlighting that LNP do not adversely affect platelet coagulability in vitro.

### Rat platelets transfected with mRNA-LNP expressed NanoLuc, circulated and localized to wound sites after transfusion into coagulopathic rodents with polytrauma

**Fig. 5.**
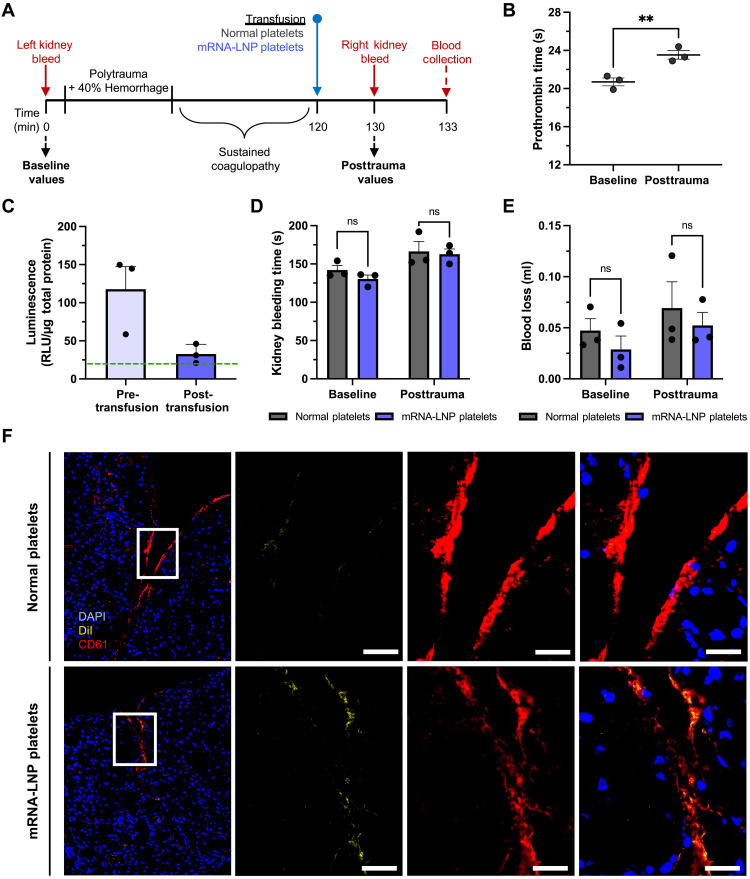
Rat platelets transfected with mRNA-LNP and transfused into rats circulated, accumulated at damaged vasculature in wounds, and contributed to hemostasis. (**A**) Overview of the transfusion procedure. Timeline not drawn to scale. (**B**) Prothrombin time in WB collected from rats receiving normal platelets before trauma (baseline) and posttrauma (*n* = 3). (**C**) NanoLuc expression in mRNA-LNP–treated platelets before transfusion and 10 min posttransfusion into rats (*n* = 3). The green dashed line represents the luminescence of the pretransfusion platelets after correcting for the predicted dilution factor of transfusing into the bloodstream. *P* values were determined by one-tailed unpaired Student’s *t* test. Values are mean ± SEM. (**D** and **E**) Kidney bleeding times (D) and blood loss (E) of rats transfused with platelets transfected with mRNA-LNP (mRNA-LNP platelets, purple) and platelets not transfected (normal platelets, gray) (*n* = 3). (**F**) Representative histological images of lacerated kidneys. mRNA-LNP–treated platelets contained a fluorescent lipid in the LNP (DiI, yellow). Sections were stained with an antibody for platelet CD61 (red) and a nuclear stain [4′,6-diamidino-2-phenylindole (DAPI), blue]. Scale bars, 20 μm.

We next examined whether mRNA-LNP–transfected platelets can contribute to hemostasis in vivo using an established rat model of polytrauma with bleeding from kidney injuries, which models acute traumatic coagulopathy with impaired platelet function (*31*). The time from injury to cessation of bleeding and the weight of blood collected throughout bleeding were measured and used as readouts of platelet function. To account for variability in clotting between animals, baseline values for kidney bleeding time and blood loss were first collected from the left kidney of each rat. This was followed by polytrauma and sustained coagulopathy, as indicated by an increased prothrombin time (Fig. 5, A and B). The right kidney was then injured, and rats were resuscitated with platelets which were either untreated (normal platelets) or transfected with NanoLuc mRNA using a rat-optimized LNP (SM-102 and POPC) labeled with the lipophilic tracer 1,1′-dioctadecyl-3,3,3′,3′-tetramethylindocarbocyanine perchlorate (DiI; fig. S14). At the termination of the experiment, blood was collected to assess the circulation time of transfected platelets by NanoLuc expression, and histology of the kidneys was assessed to determine whether transfected platelets accumulated within the injury and clot. Since NanoLuc expression was not expected to enhance nor diminish platelet function, this study specifically examined the effect of transfecting platelets with mRNA-LNP, with the expectation that there would be either decreased hemostasis or no effect on hemostasis.

NanoLuc was detected in rat platelets transfected with mRNA-LNP both before transfusion and after circulation (Fig. 5C). No loss of NanoLuc from the blood occurred within the 10 min between transfusion and blood collection. Luminescence was 3.6-fold higher in the pretransfusion platelets than in the blood after resuscitation and circulation, as the platelets were diluted by approximately 80% when transfused (*32*). Kidney bleeding time and blood volume were not statistically different between normal platelets and transfected platelets, indicating that platelets transfected with mRNA-LNP retained function in vivo (Fig. 5, D and E). Coagulation parameters and blood biochemistry were also equivalent between the two sets of resuscitated rats (fig. S15). In histological images, DiI was concentrated in the kidney wounds, indicating that transfected platelets localized to the injured tissue (Fig. 5F).

## DISCUSSION

Targeted delivery of molecules and cell therapies to damaged vasculature is a major challenge, yet platelets naturally perform this task. Despite the clinical relevance and untapped potential of platelet transfusions, genetically modifying donor platelets to enhance or “engineer” their function has not been successful. To overcome this problem, we developed platelet-optimized LNP and mRNA, resulting in successful protein expression while preserving platelet cellular function and local accumulation at damaged vasculature.

Platelet-optimized mRNA-LNP were cell specific and not optimal for transfecting the platelet precursor MEG-01 cell line. Differences in specificity between cells are largely determined by LNP characteristics such as the pKa of the ionizable lipid (*33*, *34*). A pKa below or approximately equal to the pH of the transfection buffer allows the mRNA-LNP to remain neutral before cellular uptake but adopt a greater positive charge within endosomes to enable release of mRNA into the cytoplasm (*15*). Platelet-optimized mRNA-LNP were transfected at pH 6.5, and only ionizable lipids with a pKa between 6.5 and 6.7 enabled detectable NanoLuc expression. Ionizable lipids with higher pKas lead to greater positive charge on the LNP surface, and these correlated to increased RNA uptake, increased platelet activation, and little to no protein expression. Platelets transfected with the permanently cationic lipids DOTAP and DOTMA resulted in highest Cy5 and surface CD62P intensities but no NanoLuc expression, possibly due to these cationic lipids disrupting the plasma membrane or endocytic pathways, caused by the positive surface charge of the LNP, and influencing platelet metabolic processes or inducing toxicity (*19*).

The mRNA was optimized using modified uridine bases, which enabled the expression of another reporter protein. As seen in multiple cell lines (*27*), Ψ-containing mRNA significantly increased protein expression compared to unmodified uridine. This may be from decreased immunogenicity by masking the detection from endogenous Toll-like receptors (*35*) therefore enabling higher mRNA translation. The combination of the modified mRNA and optimized LNP was used to express firefly luciferase, which has approximately 150-fold less luminescence than NanoLuc (*36*). While the levels of protein expression in platelets is lower than many other cell types, the level of expression may be sufficient to elicit a therapeutic effect in the context of an entire transfusion unit. The luciferase enzymes expressed here show that platelets can use exogenous mRNA to express enzymes that are properly folded and active. This supports that additional exogenous proteins can be expressed by platelets using the optimized mRNA-LNP. When platelets were activated by agonists it decreased subsequent NanoLuc expression, and thus, we expect that clinical applications of this technology would express the exogenous protein in platelets before transfusion, similarly to how the rat experiment was done in this study. In this manner, exogenous protein and enhanced platelet activity would be immediately present at the time of transfusion. In addition, while NanoLuc is expected to be expressed and remain in the cytoplasm, extending expression to other proteins that localize to granules or the platelet membrane is an area for future research.

Translation in platelets is both constitutive (*37*) and inducible (*12*) in response to agonists. Unexpectedly, in mRNA-LNP–transfected platelets, NanoLuc expression was not increased following platelet activation. In addition, protein expression did not correlate with surface CD62P presentation, and treatment with thrombin, CRP-XL, and ADP significantly decreased NanoLuc signal in the conditions tested here. Furthermore, when platelet activation was minimized using the inhibitor PGE1, NanoLuc expression was still robust. Platelet expression of NanoLuc may therefore be occurring from a constitutive pathway, which could explain why expression decreased following activation, as resources for protein expression could be reallocated during this state toward inducible pathways. Further investigating platelet activation and its effects on exogenous mRNA translation may be important for increasing protein expression in platelets in the future, especially under conditions where inducible expression is favorable.

Transfecting donor platelets with optimized mRNA-LNP not only preserved hemostatic function and the ability to respond to agonists in vitro but also induced a mild to moderate increase in CD62P surface expression. Although increased presentation of surface CD62P is associated with platelet storage lesion, exhaustion, and reduced function over time, moderate activation does not preclude platelets from transfusion (*38*). The hemostatic function of platelets, rather than circulation time, is most pertinent for platelets transfused to treat acute bleeding in surgery, emergency, and critical care (*39*). As observed in cold-stored platelets, moderate activation of platelets can act as a “primer” to enhance agonist response, clot strength, and clot stability (*40*). For example, cold storage of apheresis platelets results in 40 to 60% surface CD62P presentation and are transfused for resuscitation in bleeding patients (*41*, *42*). The CD62P levels of platelets transfected with mRNA-LNP were lower than that of cold-stored platelets, further suggesting that transfection will not preclude mRNA-LNP–treated platelets from transfusion. Further optimization of LNP and mRNA elements, in addition to the transfection process that currently includes washing platelets, is expected to further decrease CD62P presentation.

However, activation of platelets, such as through cold storage, also results in more rapid platelet clearance from circulation, reducing the utility of prophylactic platelet transfusions in nonbleeding, thrombocytopenic patients (*43*). In most studies, we chose not to use inhibitors of platelet activation during the processing or transfection, such as prostaglandin I_2_, because these are not normally used in blood banking and transfusion processes. While platelets transfected with mRNA-LNP circulated for at least 10 min in rats, decreasing their activation may help extend circulation times. Furthermore, rat platelets transfected with mRNA-LNP were tolerated by recipient rats and localized to the kidney incision wound. Platelets maintained the exogenous protein during circulation. Together, this supports that the transfection methodology developed here is amenable to applications within and beyond acute bleeding. Determining the circulation time beyond 10 min would also help to further define the indications that would benefit from mRNA-LNP–treated platelets. Platelets genetically modified through bone marrow transplant of platelet-precursor cells are being tested in wide-ranging preclinical and clinical applications such as for thrombolysis and bleeding disorders (*7*, *44*). Donated platelets genetically engineered with mRNA-LNP may be useful for these applications in the future.

Achieving delivery of nucleic acids and exogenous translation using platelet-optimized mRNA-LNP is a considerable opportunity to broaden and engineer platelets for various clinical applications. We expect that donor platelets engineered with mRNA-LNP may be used to treat acute bleeding and can potentially be expanded for use in thrombolytics, bleeding disorders, and applications in oncology. Platelets transfected with optimized mRNA-LNP are functional, transfusable, and accumulate in damaged vasculature. Thus, we expect that further optimizing and expanding this technology can lead to more effective platelet therapies, from targeting bleeding in hematological disorders or crises to the large range of diseases that platelets modulate.

## MATERIALS AND METHODS

### Blood component production

Pooled PCs, packed red blood cells (pRBCs), and plasma were produced by the Canadian Blood Services Blood4Research Facility in Vancouver, Canada, from donors providing signed informed consent. PC was prepared using the buffy coat production method, where PCs are leukoreduced such that residual leukocyte are within the quality criteria standard defined by Canadian Blood Services, as previously described (*45*). PC was then stored for 1 day at 22°C under constant agitation (PC1200-Pro Platelet Incubator, Helmer Scientific, Noblesville, IN). pRBCs were stored at 4°C, and plasma was stored at −30°C. To produce cold-stored platelets resembling current cold-stored platelet products, industry standard Macopharma PC bags, made from polyvinyl chloride with the plasticizer butyryl trihexyl citrate (BTHC), were welded to fit 15 ml of platelets in plasma. PCs were then aliquoted into the bags and stored at 4°C without agitation for 24 hours before use. For some experiments, analogous platelet-poor plasma was obtained from platelet units by centrifugation at 2400*g* for 20 min at 22°C. WB used in the model of dilutional coagulopathy was collected in citrated Vacutainer tubes (Becton Dickinson, Franklin Lakes, NJ) from healthy donors. In some studies, presented in figs. S4 and S9, leukoreduced single-donor platelet units were used which were produced by apheresis (Trima Accel automated component collection system, Terumo BCT Inc., Lakewood, CO), from donors providing signed informed consent. Single-donor platelet units were purchased from Versiti Blood Center of Wisconsin and treated with mRNA-LNP on the same day as collection. This study was conducted in accordance with protocols approved by the University of British Columbia Ethics Committees (H21-01516 and H16-00773), the Canadian Blood Services Research Ethics Board (2021-007), and the Medical College of Wisconsin Institutional Review Board (PRO00043006).

### Platelet transfection preparation

WPs were prepared for transfection from PC units. PC was sampled through a coupling port (Fresenius Kabi, Bad Homburg, Germany) with a sterile Leur-Lok syringe (Becton Dickinson, Franklin Lakes, NJ). PC was centrifuged at 250*g* for 20 min at room temperature as described previously (*14*). The supernatant was removed, and the remaining platelet pellet was washed once in anticoagulant citrate dextrose solution, USP, Formula A (ACD-A) (Haemonetics, Boston, MA) and once in Tyrode’s-Hepes buffer (pH 6.5) (134 mM NaCl, 2.9 mM KCl, 0.34 mM NaH_2_PO_4_, 10 mM Hepes, and 5 mM d-glucose) at 250*g* for 10 min at 22°C. WPs were suspended in Tyrode’s-Hepes buffer (pH 6.5) and counted using a Sysmex XN-550 hematology analyzer (Sysmex Corporation, Kobe, Japan). For all experiments, three PC units were washed and transfected within 24 hours.

### Synthesizing mRNA in vitro

mRNA was synthesized in bulk by in vitro transcription. Briefly, DNA templates encoding a CleanCap AG bacteriophage T7 promoter site and NanoLuc or firefly luciferase coding sequence were linearized with Sap I. RNA was produced by in vitro transcription reactions containing CleanCap AG reagent and modified nucleotides (pseudouridine and 5moU) when indicated (TriLink BioTechnologies, San Diego, CA) and purified using a RNeasy Kit (Qiagen, Toronto, ON). RNA was enzymatically tailed using a posttranscriptional tailing kit (CELLSCRIPT, Madison, WI), before an additional purification. RNA purity, integrity, and tailing efficiency were monitored by agarose gel electrophoresis. CleanCap Cyanine-5 Fluc mRNA (5moU) was purchased from TriLink BioTechnologies (San Diego, CA).

### Preparing LNP containing mRNA

LNPs containing mRNA were formulated as previously described (*46*). Briefly, an ethanolic lipid mixture containing an ionizable lipid, helper lipid, cholesterol, and DMG-PEG_2000_ (50:10:38.5:1.5 mol %) was mixed via a T-junction mixer at a 1:3 ratio with an aqueous solution of 25 mM sodium acetate pH 4 containing the desired mRNA at an amine-to-phosphate ratio of 6. The resulting mixture was dialyzed [Spectra/Por 2 Dialysis Tubing 12- to 14-kDa molecular weight cut-off (MWCO), Spectrum Labs, San Francisco, CA] 500-fold against 1× phosphate-buffered saline (PBS) and then sterile-filtered through an Acrodisc 0.2-μm syringe filter (Pall Corporation, Mississauga, ON). The formulations were concentrated in Amicon 10,000-kDa MWCO ultracentrifugation units (EMD Millipore Corporation, Billerica, MA), and the RNA content and encapsulation were determined using a Quant-it Ribogreen RNA Assay Kit (Thermo Fisher Scientific, Eugene, OR). Total lipid content was determined with the Cholesterol E kit (Fujifilm Wako Diagnostics, Mountain View, CA). Particle size and polydispersity index were measured via dynamic light scattering on the Malvern Zetasizer Nano (Malvern Panalytical, Worcestershire, England).

### Treating platelets with mRNA-LNP

WPs were treated with mRNA-LNP at an optimized ratio of 12 μg of mRNA per 40 × 10^6^ platelets (fig. S16) at a concentration of 40 × 10^6^ platelets ml^−1^ in Tyrode’s-Hepes buffer (pH 6.5) and incubated at 37°C for 4 hours before further downstream processing. When treating with the commercial reagents, Lipofectamine MessengerMAX (Thermo Fisher Scientific, Carlsbad, CA) and RiboJuice (EMD Millipore Corporation, Burlington, MA), the platelets were treated with equivalent amounts of mRNA and according to the manufacturer’s recommended protocols. Following transfection, exogenous protein expression (by enzymatic assay of NanoLuc activity), mRNA uptake (by cyanine 5–conjugated mRNA), and platelet activation (by cell surface expression of CD62P) were assessed.

### Luciferase assay

Washed and treated platelets were pelleted by centrifugation at 300*g* for 10 min at room temperature before lysing in 100 μl of Glo Lysis buffer (Promega Corporation, Madison, WI). Lysates were transferred to a 96-well flat white microplate and mixed with prepared NanoLuc substrate (Promega Corporation, Madison, WI) at a 1:1 ratio. Luminescence was recorded on the Tecan Spark Multimode Microplate Reader (Paramit Corporation, Morgan Hill, CA) with an integration time of 1000 ms and zero attenuation. The total protein content in each lysate was determined using the Pierce BCA Protein Assay Kit (Thermo Fisher Scientific, Rockford, IL) according to the manufacturer’s protocol, with absorbance measured at 562 nm on the Tecan Spark Multimode Microplate Reader. NanoLuc expression was then reported as the relative luminescence units normalized to the total protein content.

### Flow cytometry

Washed and treated human platelets were incubated in Tyrode’s-Hepes buffer (pH 6.5) at 20 × 10^6^ platelets ml^−1^ with fluorescein isothiocyanate (FITC)–conjugated mouse anti-human CD42b (HIP1, 11-0429-42, Invitrogen) and phycoerythrin (PE)–conjugated mouse anti-human CD62P (AC1.2, 550561, BD Pharmingen) antibodies for 30 min at room temperature, while rat platelets were incubated with PE-conjugated mouse anti-rat CD62P (RMP-1, 148306, BioLegend) antibody instead. For studies evaluating contaminating leukocytes, platelet-rich plasma (PRP) and leukodepleted PC were stained using PE-conjugated mouse anti-human CD45 (HI30, 12-0459-42, eBioscience). FITC-conjugated mouse immunoglobulin G (IgG1) κ (P3.6.2.8.1, 11-4714-82, Invitrogen), PE-conjugated mouse IgG1 κ (MOPC-21, 556650, BD Pharmingen), and PE-conjugated mouse IgG2a κ (MOPC-173, 400212, BioLegend) were used as isotype controls. All antibodies for human platelets were diluted at a ratio of 1:25, while those for rat platelets were diluted at a ratio of 1:50. For studies evaluating anionic phospholipid exposure, platelets were stained with PE-conjugated mouse anti-human CD62P, allophycocyanin (APC)–conjugated anti-human CD42b (HIPI, 17-0429-42, Invitrogen) and FITC-conjugated annexin V (560931, BD Pharmingen) in Hepes calcium buffer [10 mM Hepes, 145 mM NaCl, 5 mM KCl, 1 mM MgSO_4_, and 2.5 mM CaCl_2_ (pH 7.4)]. Threshold gates were set by staining in Hepes EDTA Buffer [10 mM Hepes, 145 mM NaCl, 5 mM KCl, 1 mM MgSO_4_, and 5 mM Na_2_EDTA dihydrate (pH 7.4)]. Samples were further diluted 10-fold in the corresponding staining buffer following incubation and analyzed on the CytoFLEX LX Flow Cytometer (Beckman Coulter, Indianapolis, IN) or BD Accuri C6 Plus Cytometer (BD Biosciences, San Jose, CA), using a consistent gating strategy (fig. S17) with approximately 10,000 to 25,000 total events collected. All flow cytometry data were analyzed using FlowJo v10.8.1 or BD CSampler Plus Software v1.0.34.1.

### Transmission electron microscopy

Approximately 300 × 10^6^ WPs untreated or treated with mRNA-LNP, along with cold-stored platelets, were processed for TEM analysis and imaged as previously described (*47*). Briefly, the platelets were fixed with 2.5% glutaraldehyde (Ted Pella, Redding, CA) in 0.1 M phosphate buffer (pH 7.4) for about 14 min and washed three times with 0.1 M PBS. The platelets were then stained with 1% osmium tetroxide (Electron Microscopy Sciences, Hatfield, PA) in 0.1 M PBS (pH 7.4) for about 14 min, washed twice with distilled water, and stained en bloc using 2.5% (w/v) uranyl acetate for 30 min. Samples were then dehydrated by passing through a graded ethanol series (30, 50, 70, 90, and 100%) three times and twice with 100% acetone. The samples were then passed through a graded Epon resin and acetone series (25, 50, 75, and 100%) three times under vacuum and polymerized using 100% Epon resin for 24 hours in a 65°C oven chamber (Precision Systems). Sections of 70 nm were then cut using a Leica EM UC7 Ultramicrotome and mounted on formvar-coated copper slot grids. Mounted sections were stained with 2% uranyl acetate for 12 min followed by Reynolds' lead citrate for 6 min. Stained grids were imaged using a FEI Tecnai Spirit 120-kV TEM operating at an accelerating voltage of 80 kV and magnifications of ×13,000 and ×18500.

### Characterizing LNP-treated platelets by ROTEM

The effect of LNP treatment on the hemostatic profile of transfected platelets was assessed using ROTEM and a model of in vitro trauma transfusion as previously described (*48*). Diluted whole blood (DWB) was combined with a TP consisting of blood products combined at a ratio of 1 pRBC unit:1 plasma unit:1 platelet unit before addition into the ROTEM cup. WB was diluted to 20% hematocrit (L/L) using 0.9% (w/v) saline solution (pH 5.5) (Baxter Corporation, Deerfield, IL) to simulate dilutional coagulopathy before adding the TP. Platelet components used to make the TP include WPs that were untreated or treated with ALC-0315 DOPC or CL4H6 POPC LNP not containing mRNA. LNP were dosed at 500 μg lipid per 40 × 10^6^ platelets, an equivalent lipid dosage to the mRNA-LNP used in composition screens. Untreated and LNP-treated platelets were normalized to a concentration of 300 × 10^6^ platelets ml^−1^ using Tyrode’s-Hepes buffer (pH 6.5). Once prepared, TPs were spiked into hemodiluted WB at a ratio of 70% TP:30% hemodiluted WB, to model transfusion of the TP into coagulopathic patients with hemodiluted WB. The hemostatic profile was measured by ROTEM delta (Werfen, Bedford, MA). Samples composed of either WB, hemodiluted WB, or hemodiluted WB mixed with TPs and were added to the ROTEM cup to a final volume of 300 μl. Both intrinsic pathway (INTEM) and extrinsic pathway (EXTEM) assays were performed for three biological replicates. STAR-TEM and INTEM or EXTEM reagents, at a volume of 20 μl each, were added automatically by the ROTEM machine. Reactions were allowed to run for 30 min. Key parameters of the ROTEM profile reported include clot formation time, maximum clot firmness, alpha angle (measure of clotting kinetics), and clot firmness at 10 (A10) and 20 (A20) min.

### Evaluating platelet responsiveness to agonists

Platelets untreated or treated with mRNA-LNP were activated for either 30 min or 2 hours with 10 μM Chrono-Par ADP reagent (Chrono-Log, Havertown, PA), CRP-XL [5 μg ml^−1^; synthesized as previously described (*49*)], thrombin from bovine plasma (0.1 U ml^−1^; MilliporeSigma Canada Ltd., Oakville, ON), or a combination of ADP and thrombin in Tyrode’s Solution (Modified II) (pH 7.4; Boston BioProducts, Ashland, MA). For studies evaluating their response to agonists, platelets were activated 3.5 hours post-LNP treatment. For studies evaluating NanoLuc expression from activated platelets, platelets were stimulated with agonists either 30 min before or 2 hours post-LNP treatment. In all cases, after 4-hour LNP treatment, platelets were collected for flow cytometry, and the remaining sample was centrifuged at 250*g* for 10 min, with the pellet and supernatant fractions collected for the luciferase assay.

### Preparing rat platelets for transfusion

Rat donor platelets were isolated from WB, drawn by femoral artery cannulation of Sprague-Dawley rats anesthetized with 1 to 3% isoflurane. PRP was generated by centrifugation of the citrated WB at 150*g* for 10 min at 22°C with no brake. PGI_2_ and apyrase were then added to the PRP to a final concentration of 1 μM and 0.02 U ml^−1^, respectively, and platelets were isolated by centrifugation at 1000*g* for 20 min at 22°C with no brake. Platelets were resuspended to a concentration of 40 × 10^6^ platelets ml^−1^ in Tyrode’s-Hepes buffer (pH 6.5) and transfected with DiI-labeled mRNA-LNP (SM-102 POPC) encoding NanoLuc at 12 μg of mRNA per 40 × 10^6^ platelets. Platelets were incubated with mRNA-LNP for 15 min at 37°C, centrifuged at 1000g for 20 min at 22°C with no brake, and resuspended in fresh rat plasma to a final concentration of 900 to 1200 × 10^6^ platelets ml^−1^ for 3 hours at 37°C before transfusion. mRNA-LNP–treated platelets were mixed with additional untreated platelets in a ratio of 1:1 before transfusion to minimize the amount of mRNA needed.

### Rat model of polytrauma

Recipient rats (*n* = 3 per group) underwent an established model of polytrauma and hemorrhage (*31*). Rats (372 ± 11 g) were anesthetized with 1.5 to 3% isoflurane/100% oxygen through a nose cone. The left femoral artery and vein were cannulated to monitor arterial blood pressure and for PRP transfusion, respectively. A midline laparotomy was made followed by a controlled incision with a Surgicutt Bleeding Time Device (International Technidyne Corp., Edison, NJ) at the left kidney cortex to obtain baseline measurements of bleeding time and volume, determined by the amount of time from injury until the cessation of bleeding and by the difference in weight of preweighed filter paper used to collect blood, respectively. Polytrauma was then carried out comprising crush injury at liver lobes, small intestines, and leg skeletal muscle, as well as closed femur fracture. Controlled hemorrhage was carried out by continuously withdrawing blood to a lower mean arterial pressure (MAP) of 40 mmHg over at least 5 min. MAP was maintained at 40 mmHg by repeatedly withdrawing blood until 40% blood volume was removed (blood volume was estimated as 6% body weight + 0.77 ml) (*32*). Two hours after trauma, rats were resuscitated by transfusing prepared PRP units at 20% (v/v) blood volume (equivalent to approximately 2.7 to 5.15 × 10^9^ total platelets and 1.35 to 2.57 × 10^9^ transfected platelets) through venous cannula at a rate of 1 ml min^−1^. Because of the splenic release of platelets during severe blood loss, estimation of the percentage of platelets transfused versus the percent of native platelets remaining in circulation is challenging. Ten minutes following PRP transfusion, a second incision was carried out at the right kidney cortex to measure bleeding time and volume. The terminal blood sample was then collected to measure coagulation properties (non-activated thromboelastometry, prothrombin time, and activated partial thromboplastin time), and the rats were then euthanized. The heart rate and MAP of the rats were measured from baseline collection until the end of the second kidney bleed. Rat platelets were tested ex vivo for NanoLuc activity in a plate-based activity assay, as above. Samples were taken before transfection with mRNA-LNP, before transfusion, and following circulation in coagulopathic rodents. Platelets from the pre- and posttransfusion time points were isolated as above from collected WB before lysis to determine NanoLuc content. The animal study was approved by the Institutional Animal Care and Use Committee of the U.S. Army Institute of Surgical Research (protocol #A-21-018) and Medical College of Wisconsin (AUA0007763), conducted in compliance with the Animal Welfare Act, the implementing Animal Welfare Regulations, and the principles of the “Guide for the Care and Use of Laboratory Animals.”

### Histology of kidney wounds

Kidneys (left and right) were collected immediately after euthanasia. The kidney tissues were fixed in 4% paraformaldehyde overnight, followed by 20% sucrose for 24 hours. Following fixation, the kidney tissues were embedded in optimal cutting temperature compound (Tissue-Tek, Sakura Finetek USA, Torrance, CA) and frozen in liquid nitrogen for cryosectioning at 5-μm thickness. Tissue sections were probed with mouse monoclonal anti-rat CD61 (F11, 554951, BD Pharmingen) diluted at a ratio of 1:100, followed by Alexa Fluor 594–conjugated goat anti-mouse IgG secondary antibody (A-11032, Invitrogen) diluted at a ratio of 1:750. The sections were then costained with 4′,6-diamidino-2-phenylindole for nuclear staining. The sections were imaged and analyzed by fluorescence microscopy (KEYENCE, Itasca, IL).

### Statistical analysis

All experiments consist of three biological replicates, with all values expressed as mean ± the SEM. Analyses between two groups was performed using either a one-way unpaired Student’s or Welch’s *t* test, while grouped analyses were performed using a one-way analysis of variance (ANOVA) followed by a Bonferroni’s post hoc correction. Assumptions on variance were determined using an F-test or Brown-Forsythe test as appropriate, while normality was assessed via a Shapiro-Wilks test. Statistical analysis and graphs were generated using GraphPad Prism v9.0. A *P* value < 0.05, 95% confidence intervals, was considered significant.
