# Supplementary material for: Genetically engineered transfusable platelets using mRNA lipid nanoparticles
Source: Sci Adv. 2023 Dec 1;9(48):eadi0508. doi: 10.1126/sciadv.adi0508 (PMC10691771; doi:10.1126/sciadv.adi0508)
Supplement: Supplementary file 1 — Supplementary Text Figs. S1 to S17 Tables S1 to S3 [file sciadv.adi0508_sm.pdf]

Science Advances
AAAS

Supplementary Materials for

Genetically engineered transfusable platelets using mRNA lipid nanoparticles

Jerry Leung *et al.*

Corresponding author: Christian J. Kastrup, ckastrup@versiti.org

Sci. Adv. **9**, eadi0508 (2023)
DOI: 10.1126/sciadv.adi0508

This PDF file includes:

Supplementary Text
Figs. S1 to S17
Tables S1 to S3

Supplementary Text

Abbreviations

- A10 – Amplitude after 10 minutes
- A20 – Amplitude after 20 minutes
- ADP – Adenosine diphosphate
- ALC-0315 – ((4-hydroxybutyl)azanediyl)bis(hexane-6,1-diyl)bis(2-hexyldecanoate)
- ANOVA – Analysis of variance
- CFT – Clot formation time
- CRP-XL – Cross-linked collagen-related peptide
- Cy5 – Cyanine-5
- DMG-PEG_{2000} – 1,2-dimyristoyl-rac-glycero-3-methoxypolyethylene glycol-2000
- DODMA – 1,2-dioleyloxy-3-dimethylaminopropane
- DOPC – 1,2-dioleoyl-sn-glycero-3-phosphocholine
- DOPE – 1,2-dioleoyl-sn-glycero-3-phosphoethanolamine
- DOPG – 1,2-dioleoyl-sn-glycero-3-phospho-(1'-rac-glycerol)
- DOTAP – 1,2-dioleoyl-3-trimethylammonium propane
- DOTMA – 1,2-di-O-octadecenyl-3-trimethylammonium propane
- DSPC – 1,2-distearoyl-sn-glycero-3-phosphocholine
- DWB – Diluted whole blood
- EDTA – Ethylenediaminetetraacetic acid
- ES – Egg sphingomyelin
- EXTEM – Activation of the extrinsic pathway of clot formation by thromboplastin
- FLuc – Firefly luciferase
- INTEM – Activation of the intrinsic pathway of clot formation by ellagic acid
- KC2 – DLin-KC2-DMA
- LNP – Lipid nanoparticle
- MAP – Mean arterial pressure
- MC3 – DLin-MC3-DMA
- MCF – Maximum clot firmness
- MFI – Median fluorescence intensity
- mRNA – Messenger RNA
- NanoLuc – NanoLuc luciferase
- PGE1 – Prostaglandin E1
- POPC – 1-palmitoyl-2-oleoyl-glycero-3-phosphocholine
- POPE – 1-palmitoyl-2-oleoyl-sn-glycero-3-phosphoethanolamine
- POPG – 1-palmitoyl-2-oleoyl-sn-glycero-3-phospho-(1'-rac-glycerol)
- RLU – Relative luminescence units
- ROTEM – Rotational Thromboelastometry
- SEM – Standard error of the mean
- SM-102 – 9-heptadecanyl 8-((2-hydroxyethyl)(6-oxo-6-(undecyloxy)hexyl)amino)octanoate
- STAR-TEM – Recalcification reagent used in both EXTEM and INTEM tests
- TP – Transfusion package
- WB – Whole blood

- UTP – Uridine-5'-Triphosphate
- 5moU – 5-methoxyuridine
- Ψ – Pseudouridine

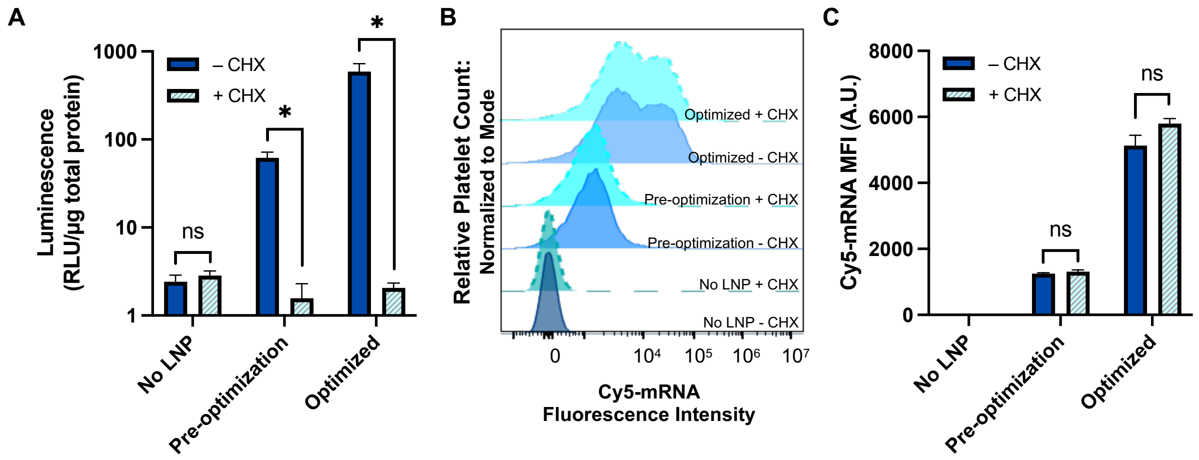

Fig. S1. Cycloheximide inhibits exogenous protein production without impacting RNA uptake. (**A**) NanoLuc expression in platelets with and without cycloheximide (CHX) (n=3). (**B**) Representative flow cytometry plots of the median fluorescence intensity (MFI) measuring fluorescently-labeled mRNA in platelets. (**C**) Graph of three replicates from flow cytometry data in panel B. P-values were determined by one-tailed unpaired Student's t-test. Data reported as mean ± SEM. ns, not significant; *P < 0.05.

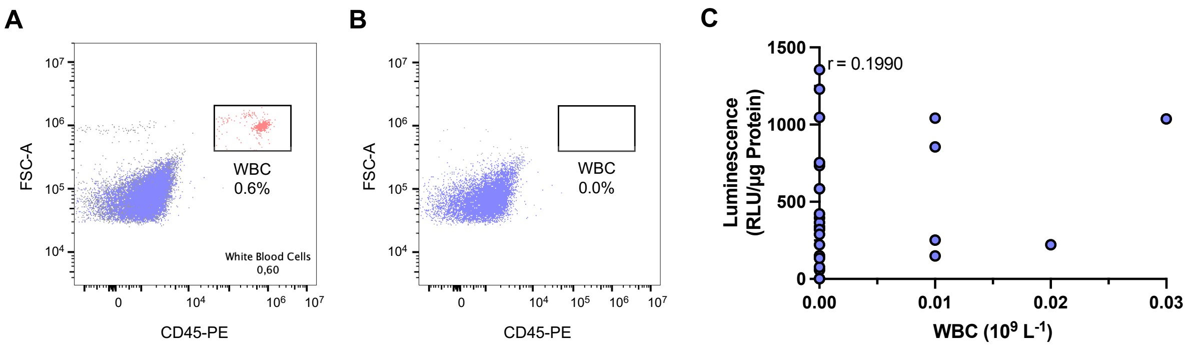

Fig. S2. Pooled platelet units are sufficiently leukodepleted. (**A** and **B)** Representative flow cytometry plots of the buffy coat fraction of whole blood (A) and pooled platelet concentrate after leukoreduction (B) stained for residual $CD45^+$ white blood cells (WBC) (red) and $CD42b^+$ platelets (light purple). (**C**) NanoLuc luminescence at various concentrations of contaminating WBC.

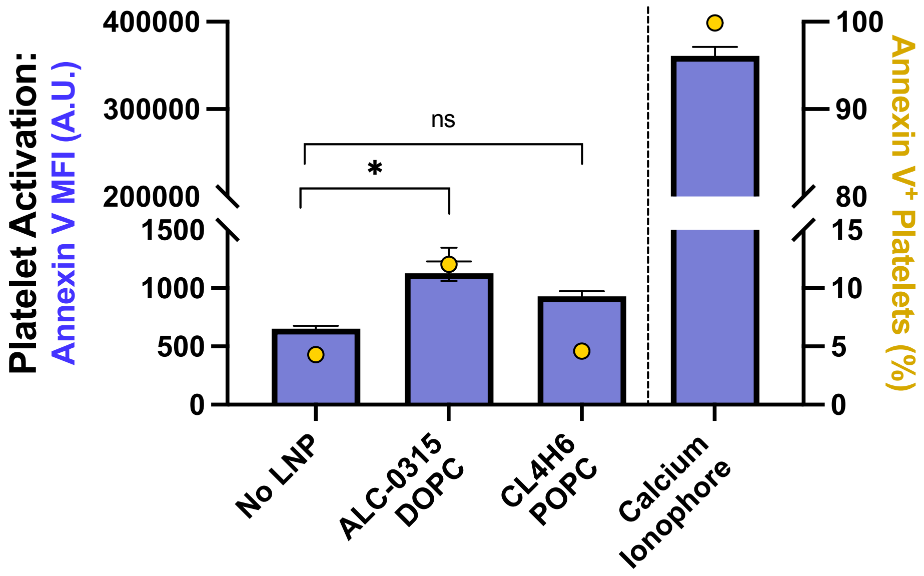

Fig. S3. Platelets treated with mRNA-LNP do not display substantial increases in annexin V binding. Quantification of the median fluorescence intensity (MFI) (bars, left y-axis) and the percentage of platelets (yellow circles, right y-axis) positive for platelet activation marker CD62P (n=3). P-values were determined by one-way ANOVA. Data reported as mean ± SEM. ns, not significant; *$P < 0.05$.

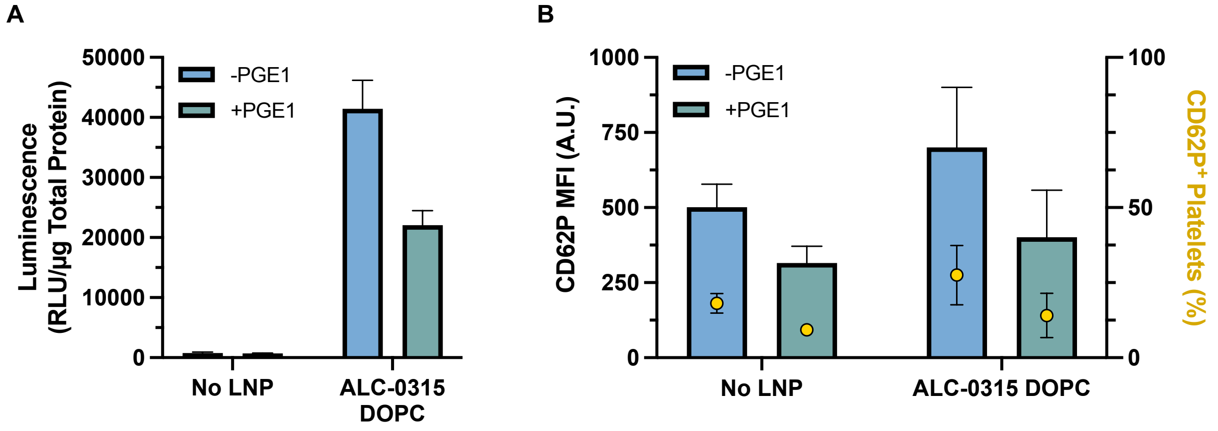

Fig. S4. Inclusion of PGE1 maintains NanoLuc expression in platelets while decreasing platelet activation. Platelets were washed with PGE1 and treated with mRNA-LNP before being assayed for NanoLuc expression (**A**) and platelet activation (**B**) measured as the mean fluorescence intensity (MFI) (bars, left y-axis) and percentage of platelets (yellow circles, right y-axis) positive for platelet activation marker CD62P (n=3). Data reported as mean ± SEM.

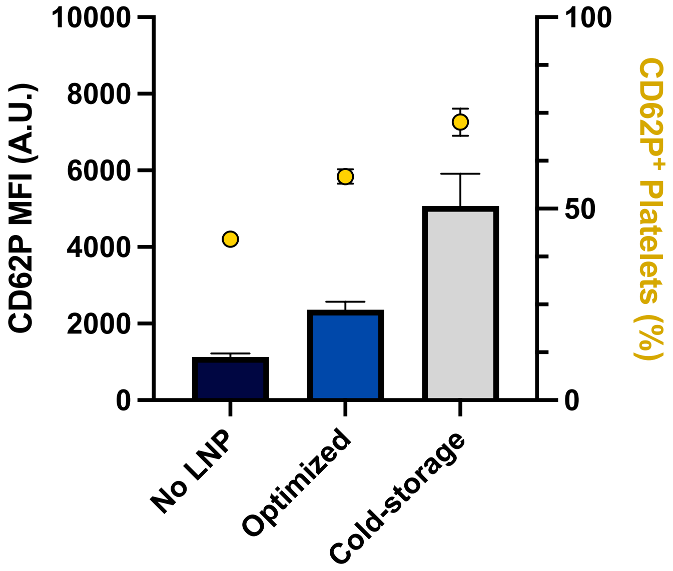

Fig. S5. Optimized LNP-treated platelets display lower surface CD62P levels than cold-stored platelets. Quantification of median fluorescence intensity (MFI) (bars, left y-axis) and percentage of platelets (yellow circles, right y-axis) positive for platelet activation marker CD62P (n=3). Data reported as mean ± SEM.

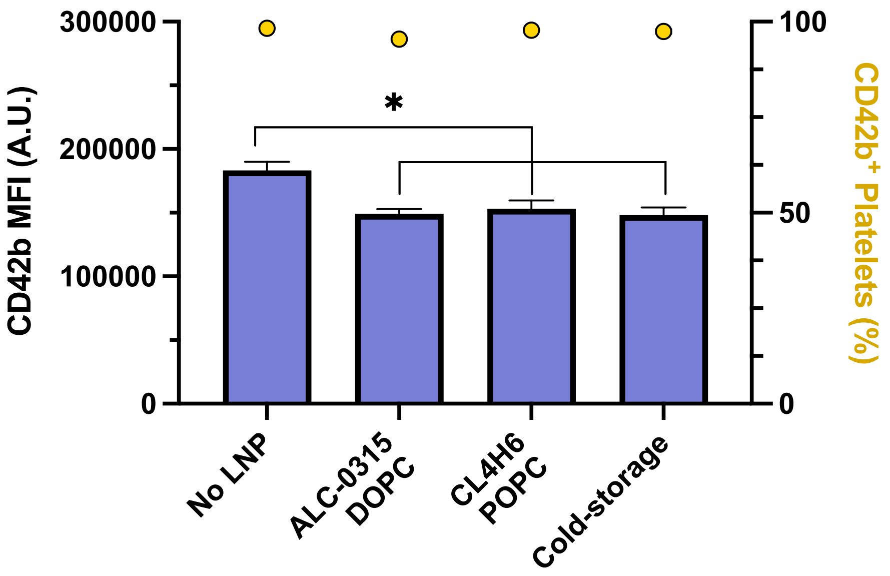

Fig. S6. CD42b levels do not substantially change when platelets are treated with mRNA-LNP and are comparable to cold-stored platelets. Quantified median fluorescence intensity (MFI) (bars, left y-axis) and percentage of platelets (yellow circles, right y-axis) positive for CD42b (n=3). P-values were determined by one-way ANOVA. Data reported as mean ± SEM. ns, not significant; *P < 0.05.

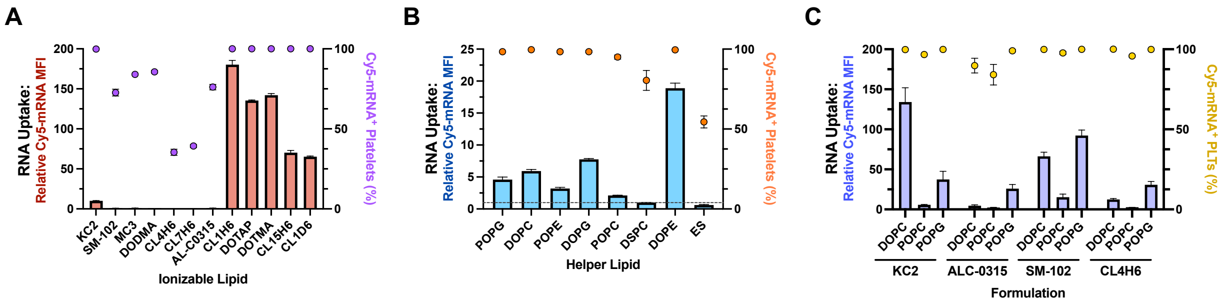

Fig. S7. Lipid composition of LNPs impacts mRNA uptake. (**A** to **C**) Relative mRNA uptake MFI (bars, left y-axes) and percentage of platelets with measurable uptake (circles, right y-axes) in screens for the ionizable lipid (A), helper lipid (B), or combination of selected ionizable and helper lipids (C) (n=3). All values were normalized to the LNP with MC3 DSPC, represented by the dashed line at values=1. Data reported as mean ± SEM.

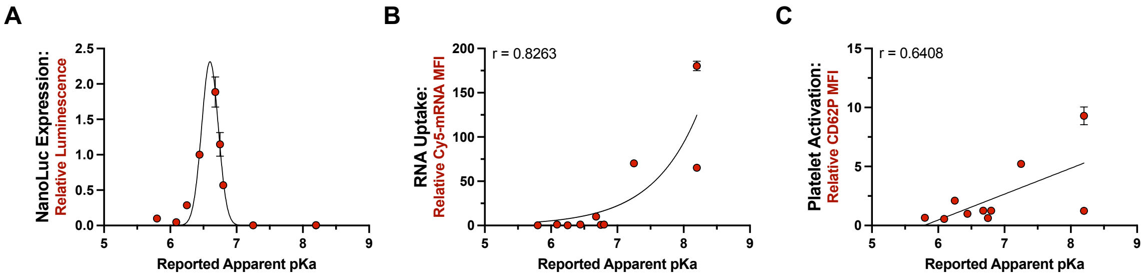

Fig. S8. NanoLuc expression, RNA uptake, and CD62P levels are dependent on the reported apparent ionizable lipid pKa. (**A** to **C**) Correlation between the ionizable lipid pKa and the relative NanoLuc expression (A), relative mRNA uptake (B), and relative platelet activation (C). All relative values were normalized to the LNP with MC3 DSPC at value=1. Data reported as mean ± SEM.

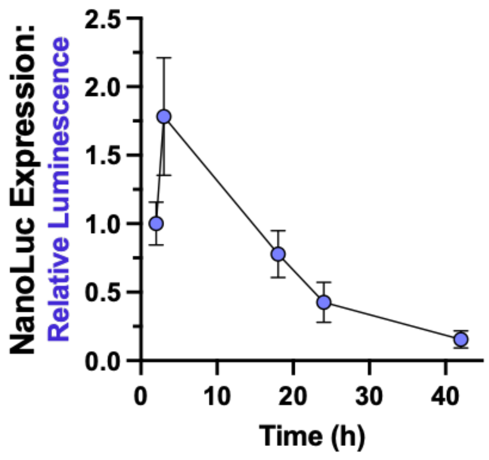

Fig. S9. NanoLuc can be detected for at least 24 h in platelets treated with mRNA-LNP. Platelets transfected with mRNA in ALC-0315 DOPC LNP were assayed up to 40 hours post-transfection for exogenous protein expression (n=3). Data reported as mean ± SEM.

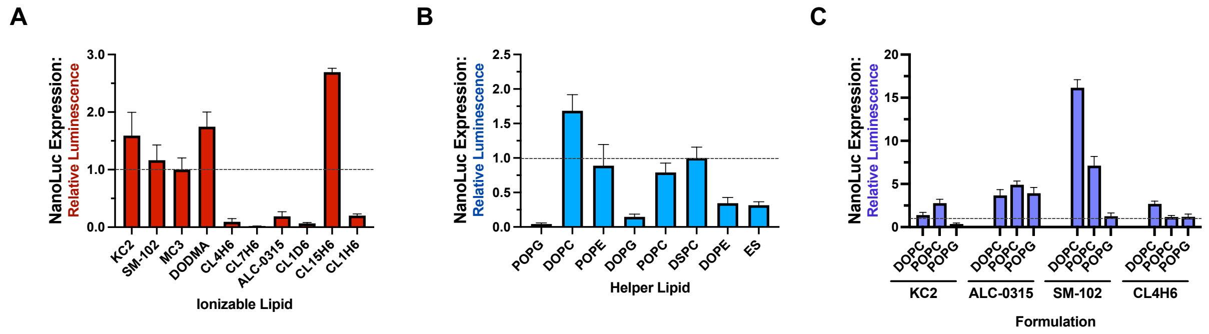

Fig. S10. NanoLuc expression in MEG-01 cells varies with lipid composition. (**A** to **C**) Relative NanoLuc expression in screens for the ionizable lipid (A), helper lipid (B), or combination of selected ionizable and helper lipids (C) (n=3). All values were normalized to the LNP with MC3 DSPC, represented by the dashed line at values=1. Data reported as mean ± SEM.

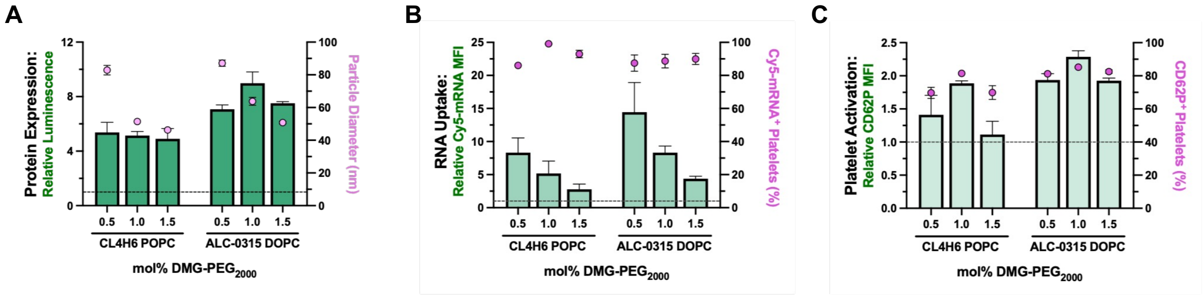

Fig. S11. Small changes in PEG concentrations do not significantly impact NanoLuc expression, but do impact mRNA uptake and platelet activation. (**A** to **C**) Relative NanoLuc expression and mean LNP diameter (A) alongside the corresponding mRNA uptake (B) and platelet activation (C) in platelets treated with CL4H6 POPC or ALC-3015 DOPC mRNA-LNP with three concentrations of PEG (n=3). All data was normalized to MC3 DSPC, represented by the dashed line at values=1. Data reported as mean ± SEM.

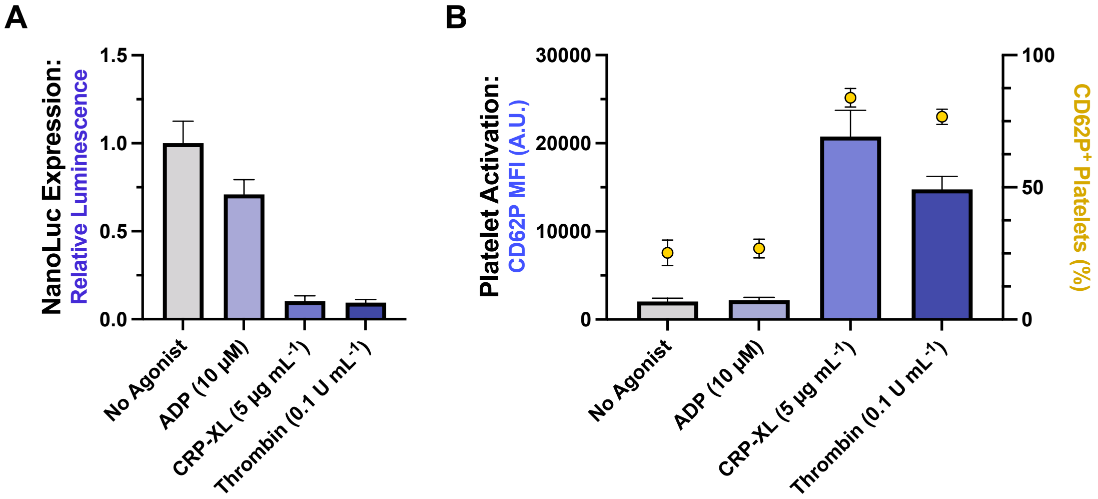

Fig. S12. Platelets stimulated with agonists prior to mRNA-LNP treatment yield decreased NanoLuc expression. (**A**) NanoLuc expression in platelets treated with agonists prior to LNP transfection, relative to NanoLuc expression in unactivated, transfected platelets (No Agonist) (n=3). (**B**) Quantification of median fluorescence intensity (MFI; bars, left y-axis) and percentage of platelets (yellow circles, right y-axis) positive for platelet activation marker CD62P in platelets treated with agonists prior to LNP transfection (n=3). Data reported as mean ± SEM.

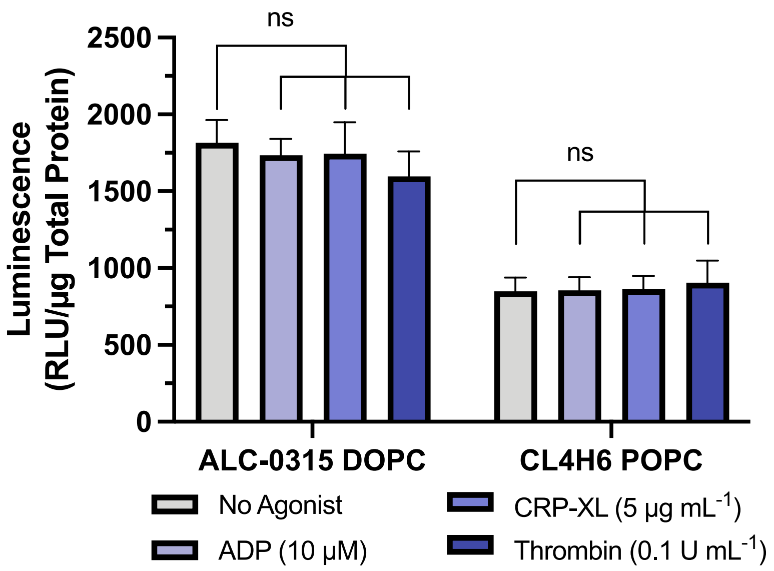

Fig. S13. Platelets stimulated with agonists 3.5 h post-mRNA-LNP treatment did not yield increased NanoLuc expression. P-values were determined by one-way ANOVA. Data reported as mean ± SEM (n=3). ns, not significant.

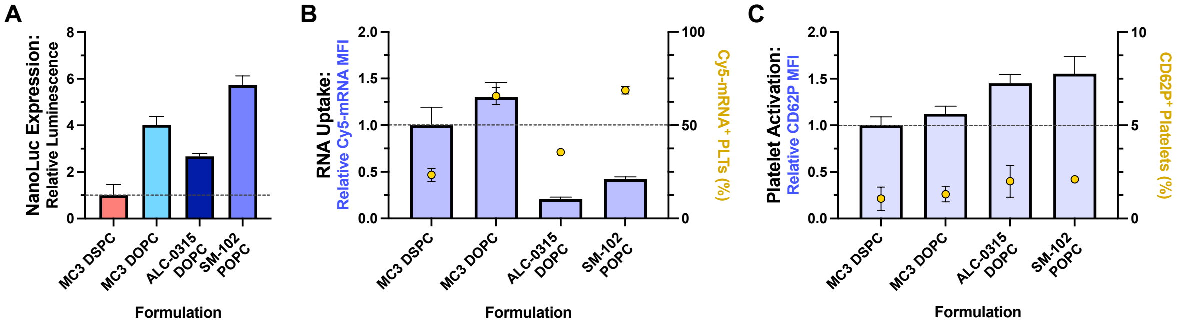

Fig. S14. SM-102 POPC LNP is optimal for transfecting rat platelets. (**A** to **C**) Relative NanoLuc expression (A), platelet activation (B), and RNA uptake (C) of rat platelets treated with different mRNA-LNP formulations. All relative values were normalized to MC3 DSPC (dashed line, left y-axes) (n=3). (**B** and **C**) Quantification of median fluorescence intensity (MFI) (bars, left y-axes) and percentage of platelets (yellow circles, right y-axes) positive for platelet activation marker CD62P (B) or Cy5-labeled mRNA (C). Data reported as mean ± SEM.

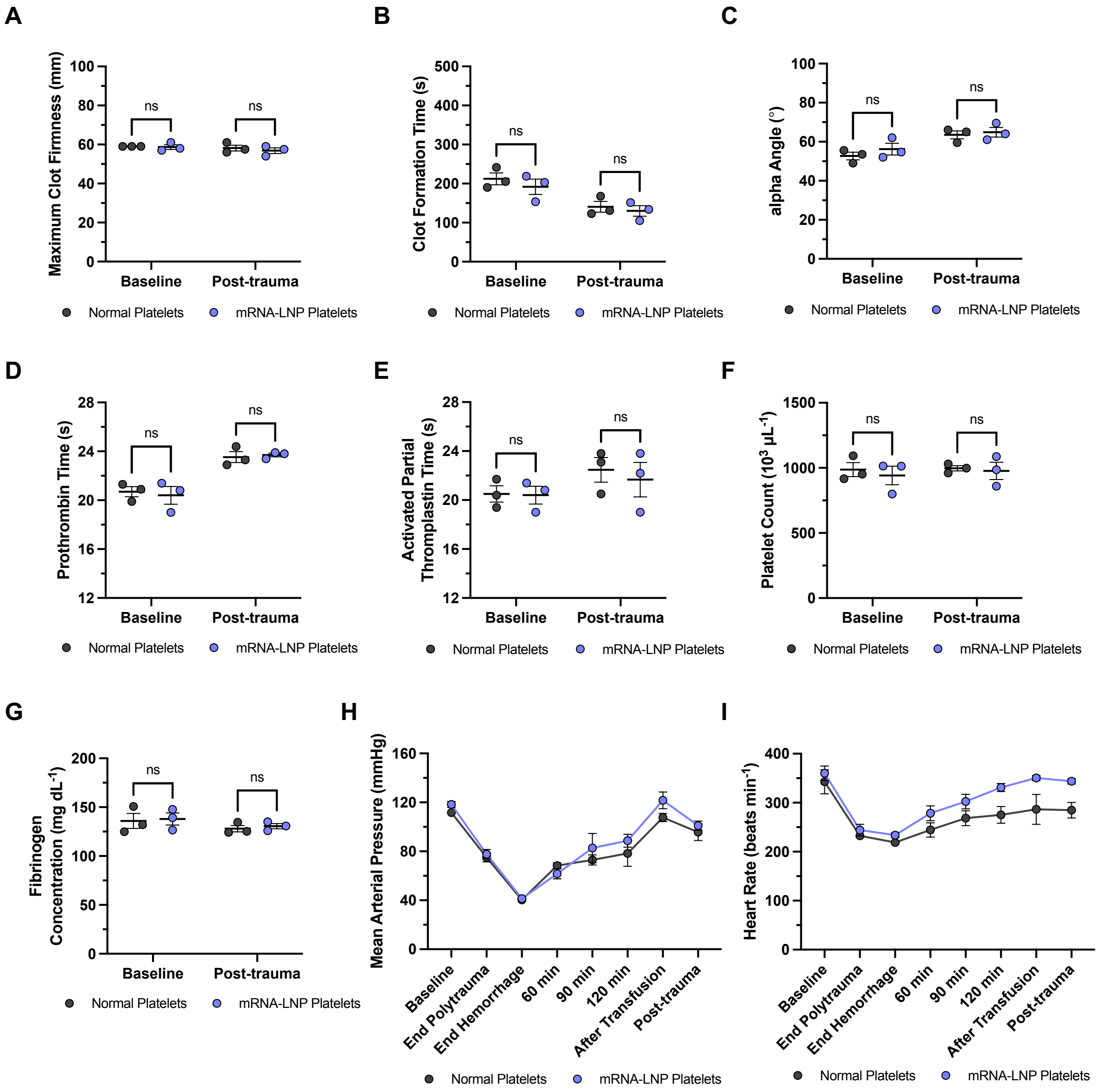

Fig. S15. In blood collected from rats transfused with platelets treated with mRNA-LNP, there are no significant differences in ROTEM coagulation parameters nor hemodynamic properties. (**A** to **I**) Plots of the maximum clot firmness (A), clot formation time (B), alpha angle (C), prothrombin time (D), activated partial thromboplastin time (E), platelet count (F), fibrinogen concentration (G), mean arterial pressure (H), and heart rate (I) (n=3). Data reported as mean ± SEM.

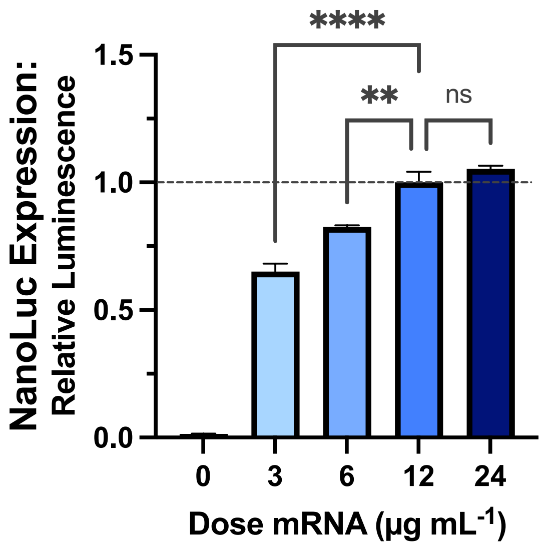

Fig. S16. Platelet transfection is dose-responsive, with maximal expression of NanoLuc at 12 μg mL^{-1} mRNA. All data was normalized to the dose of 12 μg mL^{-1} mRNA, represented by the dashed line at values=1 (n=3). P-values were determined by one-way ANOVA. Data reported as mean ± SEM. ns, not significant; **P < 0.01; ****P < 0.001.

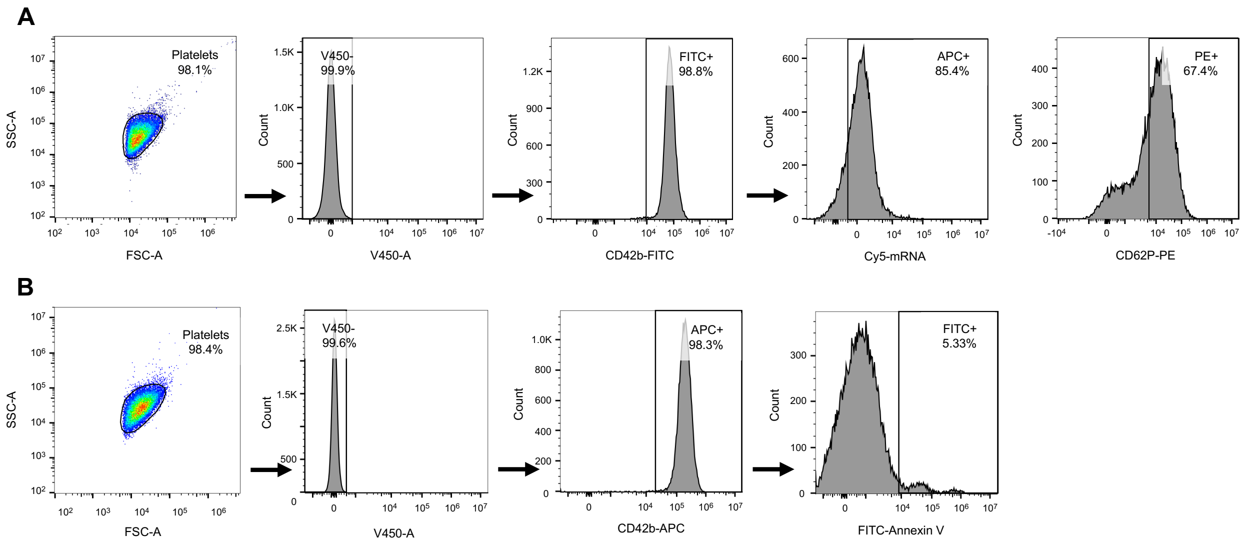

Fig. S17. Representative gating strategy for flow cytometry analysis. (A and **B)** Platelets were defined by a gate encircling 98% of the events in the forward (FSC-A) and side scatter (SSC-A) channels. The platelets were then gated for events negative in the violet 450 channel to exclude autofluorescent events, and further gated for events positive for CD42b. This population was then analyzed for events positive for CD62P and Cy5-mRNA in the PE and APC channels, respectively (A) or events positive for annexin V in the FITC channel (B). Gates set for CD42b-FITC and CD62P-PE were based on the respective IgG controls. The gate set for annexin V was based on a stained EDTA control. The gates set in the violet 450 channel and for Cy5-mRNA were based on unstained platelet controls.

LNP Formulation			Mean Particle Diameter ± SEM (nm)	Mean PDI ± SEM	Encapsulation (%)
Ionizable Lipid	Helper Lipid	DMG-PEG$_{2000}$ (mol %)			
MC3	DSPC	1.5	38.73 ± 0.45	0.109 ± 0.010	93.02
MC3	DOPE	1.5	45.11 ± 0.97	0.086 ± 0.009	89.36
MC3	DOPC	1.5	42.32 ± 0.47	0.120 ± 0.009	89.72
MC3	DOPG	1.5	43.86 ± 0.45	0.091 ± 0.006	85.14
MC3	POPE	1.5	41.23 ± 1.15	0.147 ± 0.005	93.54
MC3	POPC	1.5	40.79 ± 1.33	0.170 ± 0.006	92.43
MC3	POPG	1.5	37.99 ± 1.13	0.153 ± 0.007	87.28
MC3	ES	1.5	34.84 ± 0.63	0.132 ± 0.007	93.90
KC2	DSPC	1.5	40.99 ± 1.07	0.071 ± 0.008	91.42
KC2	DOPC	1.5	45.00 ± 1.28	0.097 ± 0.009	93.68
KC2	POPC	1.5	44.67 ± 0.39	0.104 ± 0.008	90.82
KC2	POPG	1.5	51.18 ± 0.97	0.069 ± 0.014	88.89
DODMA	DSPC	1.5	38.47 ± 1.01	0.098 ± 0.010	90.61
ALC-0315	DSPC	1.5	47.15 ± 0.94	0.061 ± 0.008	85.10
ALC-0315	DOPC	1.5	50.63 ± 0.42	0.094 ± 0.002	77.57
ALC-0315	DOPC	1	63.79 ± 2.43	0.092 ± 0.012	81.45
ALC-0315	DOPC	0.5	87.12 ± 1.92	0.159 ± 0.011	81.18
ALC-0315	POPC	1.5	44.19 ± 0.37	0.078 ± 0.003	80.09
ALC-0315	POPG	1.5	55.74 ± 1.96	0.081 ± 0.009	75.26
SM-102	DSPC	1.5	45.52 ± 0.59	0.070 ± 0.006	90.59
SM-102	DOPC	1.5	51.35 ± 0.37	0.092 ± 0.008	88.70
SM-102	POPC	1.5	45.05 ± 0.73	0.096 ± 0.003	89.08
SM-102	POPG	1.5	51.58 ± 1.01	0.068 ± 0.005	85.54
CL1D6	DSPC	1.5	38.55 ± 1.02	0.180 ± 0.002	96.37
CL1H6	DSPC	1.5	39.51 ± 0.48	0.149 ± 0.006	95.41
CL4H6	DSPC	1.5	45.31 ± 0.86	0.072 ± 0.008	87.03
CL4H6	DOPC	1.5	48.76 ± 1.22	0.104 ± 0.012	79.46
CL4H6	POPC	1.5	46.32 ± 0.30	0.086 ± 0.002	84.20
CL4H6	POPC	1	51.46 ± 1.35	0.106 ± 0.004	85.34
CL4H6	POPC	0.5	82.86 ± 2.93	0.152 ± 0.008	83.21
CL4H6	POPG	1.5	38.64 ± 0.99	0.108 ± 0.002	79.21
CL7H6	DSPC	1.5	42.22 ± 0.34	0.061 ± 0.010	70.65
CL15H6	DSPC	1.5	48.88 ± 1.14	0.074 ± 0.008	93.68

Table S1.

Mean particle diameter, polydispersity index (PDI), and percentage encapsulation for all the mRNA-LNP used in this study.

Extrinsic Pathway ROTEM Parameters					
Samples	CFT (s)	Alpha (°)	A10 (mm)	A20 (mm)	MCF (mm)
Control					
Whole Blood (WB)	116.67 ± 4.67	66.67 ± 0.67	47.67 ± 0.88	55.00 ± 0.58	56.33 ± 0.88
Diluted WB (DWB)	264.00 ± 5.69	52.00 ± 0.58	29.00 ± 0.00	35.67 ± 0.33	38.67 ± 0.33
Samples: DWB + TP (pRBC + Plasma + Sample)					
Platelets, Untreated	176.67 ± 5.78	57.67 ± 0.67	38.00 ± 0.58	46.33 ± 0.33	50.33 ± 0.33
Platelets, ALC-0315 DOPC	218.33 ± 4.91	53.00 ± 0.58	33.67 ± 0.88	41.67 ± 0.88	45.00 ± 1.00
Platelets, CL4H6 POPC	205.33 ± 1.45	54.33 ± 0.33	34.33 ± 0.33	42.67 ± 0.33	46.00 ± 0.00
Buffer, Untreated	441.67 ± 20.37	43.00 ± 0.58	23.00 ± 0.58	29.00 ± 0.58	31.67 ± 0.67
Buffer, ALC-0315 DOPC	418.00 ± 22.00	41.67 ± 1.76	23.67 ± 0.67	30.33 ± 0.33	33.00 ± 0.58
Buffer, CL4H6 POPC	416.00 ± 21.13	43.00 ± 2.31	24.00 ± 0.58	30.67 ± 0.67	33.00 ± 0.58

Intrinsic Pathway ROTEM Parameters					
Sample	CFT (s)	Alpha (°)	A10 (mm)	A20 (mm)	MCF (mm)
Control					
Whole Blood (WB)	124.33 ± 15.45	66.00 ± 2.65	46.00 ± 1.53	52.67 ± 1.33	53.33 ± 1.20
Diluted WB (DWB)	254.33 ± 2.33	54.00 ± 0.58	29.33 ± 0.33	34.33 ± 0.33	35.00 ± 0.58
Samples: DWB + TP (pRBC + Plasma + Sample)					
Platelets, Untreated	129.67 ± 1.86	66.00 ± 0.58	41.67 ± 0.33	48.33 ± 0.33	49.67 ± 0.33
Platelets, ALC-0315 DOPC	157.67 ± 9.94	62.67 ± 0.88	37.67 ± 1.33	43.67 ± 1.86	44.67 ± 1.86
Platelets, CL4H6 POPC	142.67 ± 1.76	65.00 ± 0.58	39.33 ± 0.33	44.67 ± 0.67	45.67 ± 0.67
Buffer, Untreated	393.00 ± 16.26	49.00 ± 2.65	24.67 ± 0.67	30.33 ± 0.88	31.33 ± 0.88
Buffer, ALC-0315 DOPC	447.67 ± 24.21	42.67 ± 0.33	23.33 ± 0.67	28.67 ± 0.33	29.67 ± 0.33
Buffer, CL4H6 POPC	400.00 ± 7.81	48.33 ± 1.20	24.33 ± 0.33	30.33 ± 0.33	31.33 ± 0.33

Table S2.

Rotational thromboelastometry extrinsic and intrinsic pathway parameters. Transfusion packages (TP) consisting of packed red blood cells (pRBC) and fresh frozen plasma (FFP) combined with different platelet samples were spiked into diluted whole blood (DWB). Clot formation time (CFT), alpha angle (°), amplitude at 10 minutes (A10), amplitude at 20 minutes (A20), and maximum clot firmness (MCF) reported as mean ± SEM of three biological replicates.

Extrinsic Pathway ROTEM Parameters					
Samples	CFT (s)	Alpha (°)	A10 (mm)	A20 (mm)	MCF (mm)
Whole blood (WB)	119 [94-144]	67 [62-71]	50 [42-57]	58 [50-65]	60 [52-67]
WB + ALC-0315 DOPC	83 [73-93]	73 [71-75]	56 [53-59]	63 [60-66]	64 [61-67]
WB + CL4H6 POPC	79 [76-82]	74 [73-75]	57 [56-58]	64 [63-65]	66 [65-67]

Table S3.

Rotational thromboelastometry extrinsic pathway parameters of whole blood (WB) alone or in combination with 115 µg mL^{-1} total lipid of the mRNA-LNP, ALC-0315 DOPC or CL4H6 POPC. Clot formation time (CFT), alpha angle (°), amplitude at 10 minutes (A10), amplitude at 20 minutes (A20), and maximum clot firmness (MCF) reported as mean of two technical replicates, with the range of values denoted in square brackets.
